# Comparative Phenotypic and Agronomic Assessment of Transgenic Potato with 3*R*-Gene Stack with Complete Resistance to Late Blight Disease

**DOI:** 10.3390/biology10100952

**Published:** 2021-09-23

**Authors:** Arinaitwe Abel Byarugaba, Gerald Baguma, Douglas Mutebi Jjemba, Aharinta Kenneth Faith, Arthur Wasukira, Eric Magembe, Anne Njoroge, Alex Barekye, Marc Ghislain

**Affiliations:** 1 National Agricultural Research Organisation, Entebbe P.O. Box 295, Uganda; bagumagerald@gmail.com (G.B.); jdmutebi@gmail.com (D.M.J.); aharinta@gmail.com (A.K.F.); awasukira@gmail.com (A.W.); alex.barekye@naro.go.ug (A.B.); 2 International Potato Center (CIP), P.O. Box 25171, Nairobi 00603, Kenya; e.magembe@cgiar.org (E.M.); annienjoroge@gmail.com (A.N.)

**Keywords:** genetic engineering, potato, late blight disease, regulatory trials, yield, agronomic performance

## Abstract

**Simple Summary:**

A potato transgenic event was shown to have complete resistance to late blight, and no other biologically significant differences with the original variety it derives from. Field research, carried out at three locations for three seasons, demonstrates the value this transgenic potato events can deliver to potato farmers in Africa.

**Abstract:**

Transgenic potato event Vic.172, expressing three naturally occurring resistance genes (*R* genes) conferring complete protection against late blight disease, was evaluated for resistance to late blight, phenotypic characterization, and agronomic performance in field conditions at three locations during three seasons in Uganda. These trials were conducted by comparison to the variety Victoria from which Vic.172 derives, using identical fungicide treatment, except when evaluating disease resistance. During all seasons, the transgenic event Vic.172 was confirmed to have complete resistance to late blight disease, whereas Victoria plants were completely dead by 60–80 days after planting. Tubers from Vic.172 were completely resistant to LB after artificial inoculation. The phenotypic characterization included observations of the characteristics and development of the stems, leaves, flowers, and tubers. Differences in phenotypic parameters between Vic.172 and Victoria were not statistically significant across locations and seasons. The agronomic performance observations covered sprouting, emergence, vigor, foliage growth, and yield. Differences in agronomic performance were not statistically significant except for marketable yield in one location under high productivity conditions. However, yield variation across locations and seasons was not statistically significant, but was influenced by the environment. Hence, the results of the comparative assessment of the phenotype and agronomic performance revealed that transgenic event Vic.172 did not present biologically significant differences in comparison to the variety Victoria it derives from.

## 1. Introduction

The potato (*Solanum tuberosum* L.) is the third most important food crop globally [1]. In parts of East and Central Africa (Burundi, D.R. Congo, Kenya, Rwanda, and Uganda), it is one of the most important food and cash crops. It is grown by over 480,000 smallholder farmers in Uganda [2]. The eighth largest potato producer in sub-Saharan Africa, Uganda has an average annual production of 400,000 tons harvested from an average of 100,000 ha [3,4]. Production is concentrated in highland areas, primarily the southwestern highlands of Kabale, Kisoro, and Kanungu districts, which produce about 87% of the country’s total output [5].

Potato productivity is estimated on average to be in the range of 4 t/ha in Uganda and 8.3 t/ha in eastern Central Africa, which is well below the global average of 21 t/ha [6]. However, such a yield is easily achievable on research stations under good crop management practices in Uganda [7]. Several factors have been associated with low potato productivity in Uganda, one of which is varietal susceptibility to late blight (LB) disease caused, by *Phytophthora infestans,* which is responsible for 13–60% of yield loss. Without timely application of fungicides, LB disease can even lead to complete yield loss [8,9].

Potato varieties with resistance to late blight have been developed by conventional breeding, but rapidly lose their resistance due to the emergence of virulent isolates. Breeders have used wild *Solanum* species as a source of high levels of resistance, the first of which was *Solanum demissum* [10]. However, the pathogen has overcome the resistance provided by these single *R* genes through strains that lack the pathogen protein (effector) recognized by the R protein, which triggers host plant resistance. Exploiting sources of resistance to disease from wild species through conventional breeding is an extremely slow process, and there are major limitations due to inbreeding depression and polyploidy [11]. For instance, the 2005 release of the varieties Bionica and Toluca with LB resistance from *S. bulbocastanum,* including the *Rpi-blb2* gene, came 46 years from the first cross [12].

Single *R* genes with broad-spectrum resistance to LB disease have been introduced into potato varieties that were released commercially in the U.S. [13,14,15,16,17]. However, stacking of *R* genes with known efficacy has been recognized as most effective strategy to control LB disease [18,19,20]. The direct transfer by genetic engineering techniques of a stack of multiple *R* genes from the potato’s wild relatives into potato varieties has been shown to confer complete resistance to LB disease [21,22,23]. After transforming potato varieties grown in Africa with a stack of three *R* genes (*RB* and *Rpi-blb2* from *S. bulbocastanum* and *Rpi-vnt1.1* from *S. venturii*), a dozen of transgenic events was evaluated in confined field trials and proved to have complete resistance to LB disease [24].

One of those 3*R* varieties, Victoria, is the most popular variety in Uganda and is also grown in other countries of east and central Africa. Following molecular characterization and resistance bioassays, we selected the potato transgenic event Vic.172, derived from the variety Victoria, as a candidate for future release. To this end, risk assessment must be conducted towards the demonstration that the transgenic event does not show significant weediness in the natural environment and is as safe as the original variety regarding human and animal health. Such regulatory-demanded assessments include phenotypic characterization and agronomic performance in confined field trials at multiple locations over several seasons [25]. These regulatory trials aim to identify genotypic differences between the transgenic event (Vic.172) and its nearest isogenic line (the variety Victoria) that persist across locations and seasons, and whether these differences pose a threat to the environment and human or animal health.

Differences in potato performance can have varied origins. Seed tuber quality is a well-known source of variation in agronomic performance due to small physiological differences between seed tubers and/or the accumulation of pathogens [26]. Tissue culture, and in particular regeneration from tissue culture, is known to cause genetic changes affecting the phenotype and agronomic performances of potato transgenic events [27,28]. In potato, somatic mutations have also been known to lead to new variants of existing potato cultivars [29]. The epigenome seems to be affected by tissue culture, which suggests that such changes may be reversible over time [30]. One of the oldest potato varieties, Russet Burbank, released in 1914, has led to the selection of variants cultivated in different states of the US and still has potential for improvement through selection of somaclonal mutants [31]. This study focused on the evaluation of the phenotype and agronomic performance of the transgenic event Vic.172 relative to the variety Victoria across environments representing three different agroecological zones in Uganda. The study aimed to assess whether there are any biologically significant differences between the transgenic event Vic.172 and the variety Victoria when grown under identical field conditions.

## 2. Materials and Methods

### 2.1. Plant Materials

The test genotypes were transgenic event Vic.172 and the non-transgenic variety Victoria from which the transgenic event Vic.172 was derived. The variety Victoria was released in 1991 from the CIP breeding line CIP381381.20 and was originally an LB-resistant variety. In a 2007 survey of breeders and other experts, adoption of the potato variety ‘Victoria’ in Uganda was estimated at 35% [32]. However, it declined soon afterwards due to its increased susceptibility to LB disease [8]. The transgenic event Vic.172 was produced by genetic transformation of the CIP breeding line CIP381381.20 using the pCIP99 binary transformation vector bearing three resistance (*R*) genes (*RB*, *Rpi-blb2* from *S. bulbocastanum*, and *Rpi-vnt1.1* from *S. venturii*) and the hyper-virulent strain EHA105 of *Agrobacterium tumefaciens* [24]. Molecular characterization revealed that it contains a single copy of intact T-DNA for the 3 *R* genes, no vector backbone sequences, and it was found to be inserted into a non-coding region of the potato genome (manuscript in preparation).

### 2.2. Experimental Sites

**Figure 1:**
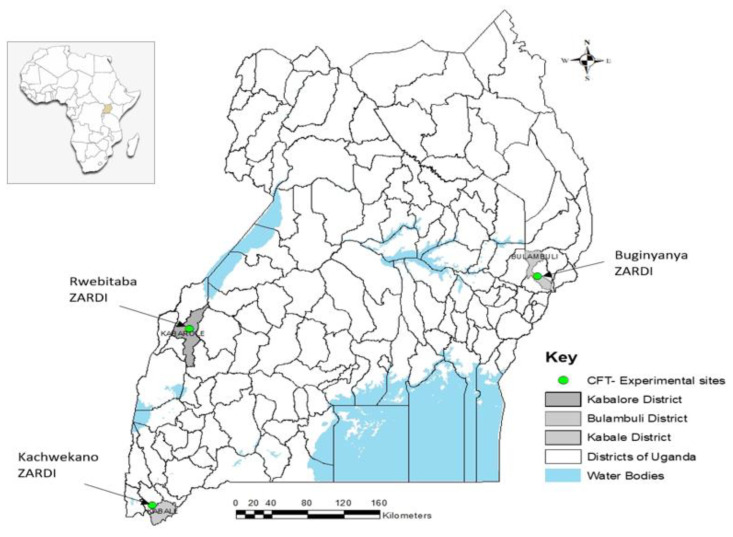
Location of the three experimental sites in Uganda, representing the main agroecologies for potato production, where confined field trials were undertaken.

The study was conducted in confined field trials (CFT) at three locations with differing edaphic and climatic conditions that are known to favor potato growth in Uganda (Figure 1). (1) Kachwekano Zonal Agricultural Research and Development Institute (ZARDI) is located in Kabale district, at an altitude 2225 m.a.s.l., in the southwestern highland agroecological zone, which is cool and windy. The rainfall pattern is bimodal with a short rainy period in the first growing season (March to June) and a long rainy period during the second season (October to January). The soils are fertile and sandy loams. (2) Rwebitaba ZARDI, located in Kabarole district, at an altitude 1531 m.a.s.l., has a warmer climate but experiences a similar bimodal rainfall pattern. The site has loamy soils with a coarse texture. (3) Buginyanya ZARDI, located in Bulambuli district, at an altitude of 1887 m.a.s.l., belongs to the southeastern highland agroecological zone with bi-modal rainfall. The site’s soils are dominated by ferrisols. These locations constitute the three environments in which data were collected to assess phenotype and agronomic performance.

### 2.3. Experimental Design

One hundred tubers of each of the transgenic event Vic.172 and the variety Victoria were planted per plot and replicated three times with a spacing of 75 × 30 cm using a completely randomized block design (CRBD) at each of the three sites. Three blocks were used at each site with plot size of 3 × 3 m per replicate per genotype. Plots were separated from each other by 1 m. Straight-line trenches about 10 cm deep were dug and 100 kg/ha of nitrogen (NPK 17:17:17) was applied using hands. Sprouted tubers were placed into the trenches with sprouts facing upwards, and were then covered with enough soil to form mounds over the tubers. Standard crop management practices were followed, such as weeding and pest control for cutworms, aphids, and leaf miners. Weeding was conducted manually 4–5 times per season whereas insect pest management was conducted 2–3 times per season using the systemic insecticide Rocket^®^ (profenofos 40% + cypermethrin 4%). The plots protected from late blight were sprayed weekly with fungicides, alternating between Ridomil Gold^®^ (metalaxyl-M 40 g/kg; mancozeb 640 g/kg) and mancozeb (mancozeb 80% *w*/*w*), depending on the weather conditions at each site. Ridomil Gold^®^ was applied at a rate of 2.5 kg/ha while mancozeb was applied at a rate of 2.1 kg/ha. This experimental design was used at all locations during the two seasons of the multi-location trials. The first trial took place at a single location (Kachwekano ZARDI) during the first season (March–June 2019) to demonstrate trait efficacy (no fungicide treatment) and data collected is referred to as CFT-5. Data for all three locations in the second season (October 2019 to January 2020) is referred to multi-location CFT-3 (ML-CFT-3). Data collected in the third season (October 2020 to January 2021) is referred to as ML-CFT-4-Trial-1 and ML-CFT-4-Trial-2 because there were two identical trials at each location. Trial design in the multi-location trials included trait efficacy trials (no fungicide treatment on any plots) and regulatory trials (with fungicide treatment on all plots). Though the transgenic event Vic.172 does not require fungicide protection, the same fungicide treatment was applied on both the plots of transgenic event Vic.172 and the variety Victoria to have the identical growth conditions needed for comparative analyses. Data collected from these regulatory trials were used for phenotypic characterization and agronomic performance.

### 2.4. Late Blight Disease Scoring

Development of late blight on leaves was monitored on a weekly basis in plots without fungicide treatment and disease severity and plant leaf area affected (PLAA), were scored following a standard protocol for late blight evaluation [33]. Disease progress curves were generated based on the severity rating estimated from the weekly data recording for the transgenic event Vic.172 and the variety Victoria.

Cleaned and undamaged field-grown tubers from ML-CFT-3 were shipped from the trial site in Uganda to the biosafety facilities in Kenya in 2019. Two bioassays, adapted from [34], were conducted. In the first assay, three tubers were sliced (1 cm thickness) and each slice was placed in a petri dish. The bottom side of the tuber slice was infected with a 4000 sporangia/mL inoculum. In the second, three tubers were inoculated by filling a tunnel made halfway into the tuber with the 4000 sporangia/mL inoculum. Negative controls were made for both assays using sterile water instead of the inoculum. The tubers were placed in the dark in a Conviron^TM^ chamber maintained at a relative humidity of between 80% to 100% and temperature of 18 °C for 25 days. The resistance was visually scored 23 days after inoculation based on the presence of sporulation on the tuber slices and on the tunnel sides, after cutting the tuber along the tunnel.

### 2.5. Phenotypic Characterization

Data were collected weekly from the fungicide-treated plots during the four phenological phases: plant emergence, plant and foliage growth, flower, and berry formation, and bulking of tubers. Data were recorded weekly, electronically, using a handheld personal digital assistant (PDA) (Motorola 9500 Series MC9596). All observations were carried out at the plot level by recording most frequently observed phenotype or the average value of all plant and organs observed. Stems were evaluated for their color using a 1 to 5 scale (1 = light green, 2 = green, 3 = dark green, 4 = purplish green), and wing (0 = absent; 1 = present) and were counted per plant 50 days after planting (DAP). Leaves were evaluated by the average number of leaflets per leaf, texture (1 = smooth, 2 = smooth hairy, 3 = rough, 4 = rough hairy), average number of interjected leaf numbers, color (1 = light green, 2 = green, 3 = dark green), and openness (1 = open, 2 = closed). Flowers were evaluated by estimating the average number per plant, and their color (1 = white, 2 = light purple, 3 = purple). At harvest, tubers were characterized by observing medium and large tubers for their shape, skin, and flesh color.

### 2.6. Agronomic Performance Assessment

Seed tubers for the three locations were produced in the screen house at Kachwekano ZARDI. Seed tuber sprouting was tested by keeping seed tubers in aerated plastic trays under diffused light at Kachwekano ZARDI. The seed tubers for the transgenic event Vic.172 and the variety Victoria were able to produce 3–4 sprouts within two to three months after harvesting. Tubers were ready for planting when the sprouts were 1–1.2 cm long. After planting, observations of emergence of sprouts above the soil were made weekly for each plot until plant stand was well established, around 50 DAP. All plots were sprayed with fungicide to ensure the agronomic performance of the transgenic event and the variety Victoria could be compared independently from the resistance to LB. Plant vigor was assessed 45 DAP using a 1 to 5 scale (1 = poor, 3 = average,5 = excellent). Plant and foliage growths were estimated by taking the average plant height and width in cm for each of the five rows. All data were collected at plot level.

After tuber bulking, at ~90 DAP, the above ground parts of the plants were cut off (dehaulmed) and tops were incinerated. Two weeks later, tubers were harvested and graded into three categories. The weights and numbers of tubers in each category were recorded for each plant. The following categories were used: Category I: commercial tubers/big tubers measuring > 60 mm, Category II: commercial tubers/medium tubers 30–60 mm, Category III: non-commercial/small tubers measuring < 30 mm. The number and weight of rotten tubers and tubers with defects were also counted at plot level. Yield assessment was conducted using the data on each individual plant, eliminating the outliers falling below or above the interquartile range (IQR) of the raw data and that with higher residuals, using categories 2 and 3 for marketable yield. Marketable tuber yields in kilograms per plant were converted to tons per ha by multiplying by 47,619 which is the potato density per ha in Uganda.

### 2.7. Data Analysis

The Genstat Generalized Linear Module (GLM) was used to analyze and interpret the data using the statistical package Genstat (VSN International Ltd., Hemel Hempstead, UK) [35]. Analysis of variance (ANOVA) results was used to calculate least significant differences (LSD) between the two genotypes—the transgenic event Vic.172 and the variety Victoria—at each location. For comparing separated means, LSD and pairwise comparisons tests were carried out at a probability level of *p* < 0.05 [36].

## 3. Results

### 3.1. Trait Efficacy in the Field

**Figure 2:**
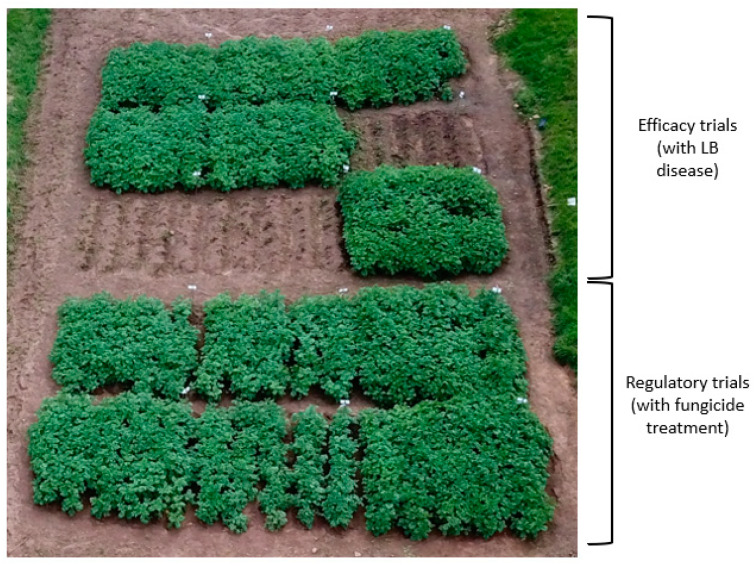
Confined field trial at Kachwekano ZARDI of plots with transgenic event Vic.172 and the variety Victoria. Efficacy trials were made of transgenic event plots (green plants) and Victoria plots (moribund plants), which were devastated by LB under natural infestation. Regulatory trials consisted of plots of the transgenic event Vic.172 and the variety Victoria under fungicide treatment (all green) during the short-rain season in 2019.

The development of late blight on leaves was monitored weekly during the confined field trial at Kachwekano ZARDI (CFT-5), at the three locations (ML-CFT-3) and again at Kachwekano ZARDI (ML-CFT-4). Under natural infestation, without fungicide treatment, the plots planted with the transgenic event Vic.172 remained green whereas the plots with the variety Victoria were completely destroyed by LB disease (Figure 2).

**Figure 3:**
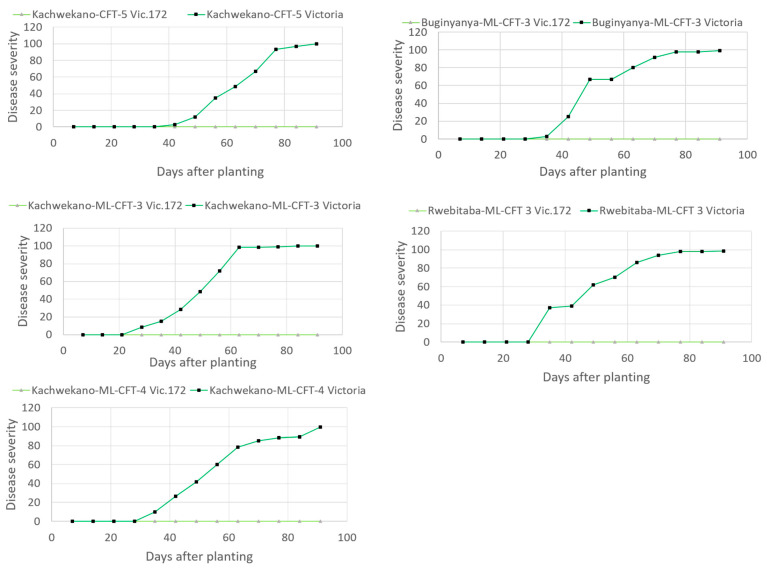
Disease progress curves of the transgenic event Vic.172 (light green) and the variety Victoria (green) at Kachwekano ZARDI during the first season of 2019 (Kachwekano-CFT-5); Buginyanya ZARDI, Kachwekano ZARDI and Rwebitaba ZARDI during the second season of 2019; (Buginyanya-ML-CFT-3, Kachwekano-ML-CFT-3, Rwebitaba-ML-CFT-30); and at Kachwekano ZARDI during the third season of 2020 (Kachwekano-ML-CFT-4). Efficacy trials were conducted without any fungicide treatment.

The disease appeared at 4–5 weeks (35 days) after planting and was observed only on the leaves of non-transgenic plants in CFT-5 and ML-CFT-3 and 4. The disease expanded rapidly on the non-transgenic varieties and reached 100% between 63 and 70 DAP for all the ML-CFT Victoria variety plants. All the transgenic plants remained clear of any late blight infection throughout the trial period (Figure 3).

Tuber resistance bioassays were conducted in Nairobi, Kenya, using tubers harvested at Kachwekano ZARDI, in Uganda. Tuber slices were inoculated as well as tubers with infected holes. After 23 days, none of the tuber slices from the transgenic event Vic.172 show sporulation whereas those from the variety Victoria did. Tubers with infected holes were halves through the hole revealing the same results as for the tuber slices (Figure S1).

### 3.2. Phenotypic Characterization

#### 3.2.1. Stems

The average number of stems per plant ranged from two to four across the three locations. The highest number of stems (4) per plant was observed at Rwebitaba for all plots (Figure S2). There were no statistically significant differences (*p* < 0.05) in the average number of stems per plant between the transgenic event Vic.172 and the variety Victoria within each location (Table S1).

Stem color was recorded as ‘4-Purplish Green’ at Kachwekano ZARDI for all plots from week 6 until 13, ‘Purplish Green’ from week 6 to 11 followed by ‘Green’ and ‘Light Green’ during senescence on week 12 and 13 at Rwebitaba ZARDI for all plots, and ‘Purplish Green’ for all pots from week 6 until 13 at Buginyanya ZARDI. Stem color differences were not observed between the transgenic event Vic.172 and the variety Victoria at any location and between locations. Fading of the purplish green hue towards senescence was observed at Rwebitaba ZARDI for both genotypes.

Stem wing was observed to be present from the third week after planting up to maturity at all three locations. It was only absent in the second week after planting across the locations for the two genotypes, as the crop had just emerged. No differences in stem wing were observed between the transgenic event Vic.172 and the variety Victoria at any location or between locations.

#### 3.2.2. Leaves

The average number of leaflets per leaf was in the range of seven to nine during weeks 6 to 9 in most of the plots at all the locations, with slight variation between weeks (Figure S3). No significant differences in number of leaflets between the transgenic event Vic.172 and the variety Victoria were detected at any location (Table S2).

Texture of the leaves was rated ‘Smooth and hairy’ for most of the time as the crop transited from one phenological stage to another at each location. For some weeks, both genotypes were rated ‘smooth’ especially when they were still young, and in a few instances were rated ‘rough and hairy’ as crop transitioned to the mature stage. Based on these observations, there were no differences in the texture of the leaves between the transgenic event Vic.172 and the variety Victoria.

The average number of interjected leaves was assessed during the growth phase (Figure S4). The results revealed more interjected leaves at Kachwekano ZARDI compared to other two locations. The maximum was observed between the weeks 7 to 9 after planting with an average of 15 interjected leaves. However, there were no statistically significant differences in the average number of interjected leaves between the transgenic event Vic.172 and the variety Victoria while the crop transitioned through the different phenological growth stages (Table S3).

Leaf color was rated ‘light green’ until maturity and then ‘Green’ for all plots at each location. There were no differences in the leaf color between the transgenic event Vic.172 and the variety Victoria at any location, nor between locations.

Leaf openness was rated closed in ML-CFT-3 and open for most of the growth period during ML-CFT-4-Trial-1 and Trial-2. There were no differences between the transgenic event Vic.172 and the variety Victoria.

#### 3.2.3. Flowers

Differences in flowering levels between the transgenic event Vic.172 and the variety Victoria were assessed based on the number of flowers observed per week during the blossom stage (Figure S5). Flowers were observed from week 7 to 12. The average number of flowers per plant ranged from 10 to 16 at Kachwekano ZARDI, 0 to 13 at Rwebitaba ZARDI, and 0 to 7 at Buginyanya ZARDI, with slightly fewer flowers on the transgenic event Vic.172 than on the variety Victoria at each location. ML-CFT-4-Trial-1 and Trial-2 had fewer flowers compared to ML-CFT-3, indicating seasonal variation in the degree of flowering across locations. Across locations, the degree of flowering in the transgenic event Vic.172 and the variety Victoria was not statistically different (Table S4).

Flower color was rated as ‘Purple’ at Kachwekano ZARDI and Rwebitaba ZARDI, and 2-Light Purple’ at Buginyanya, with no differences between the event Vic.172 and variety Victoria; no berries were observed at any of the locations.

#### 3.2.4. Harvested Tubers

There were no significant differences in tuber shape, skin color, or flesh color between the transgenic event Vic.172 and the variety Victoria at any of the three locations. All tubers were recorded as round, red skin, and white flesh.

### 3.3. Agronomic Performances

#### 3.3.1. Sprouting and Emergence

Seed tubers of the transgenic event Vic.172 and the variety Victoria sprouted 2 months after harvest at room temperature under diffused light at Kachwekano, without any physiological stress or chemicals to interrupt dormancy (Figure S6). There was no statistically significant difference between the transgenic event Vic.172 and the variety Victoria in the number of sprouts per tuber nor in sprout length by the end of 3 months (Table S5). Potato plants emerged from two to eight weeks after planting. The data show that there were no differences in emergence between the transgenic event Vic.172 and the variety Victoria at each location.

#### 3.3.2. Plant and Foliage Growth Characterization

**Figure 4:**
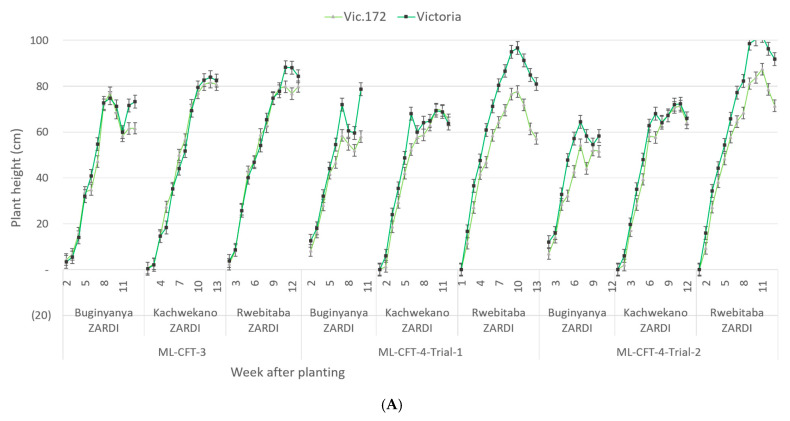

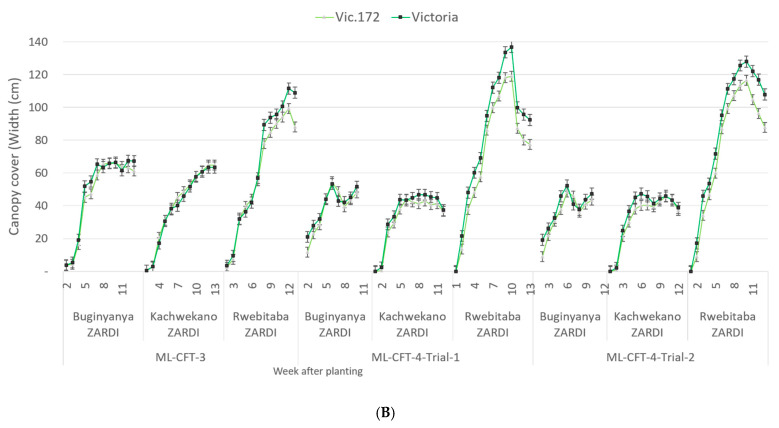
Plant growth of canopy of the transgenic event Vic.172 (light green) and the variety Victoria (green) plots at three locations during three trials measured by the average height (**A**) and width (**B**) of potato rows. Error bar represents the standard error.

After emergence, the growth of the plants within each plot was observed weekly from the second week until dehaulming. Data on canopy width and height were recorded at the plot level. The progress of plant growth measured by the average of the three plots at each location shows a similar curve for the transgenic event Vic.172 and the variety Victoria (Figure 4). All the potato plots obtained their maximum growth width and height around the 10th week after planting in each location and were deemed suitable and commercially acceptable.

#### 3.3.3. Plant Vigor

Plant vigor was rated ‘excellent’ for most of the plots at all locations during ML-CFT-3 and were rated ‘Average’ to ‘excellent’ during ML-CFT-4-Trial-1 and Trial-2 with no significant differences between the transgenic event Vic.172 and the variety Victoria.

#### 3.3.4. Yield Estimation

##### Marketable Yield of Both Genotypes across Locations

**Figure 5:**
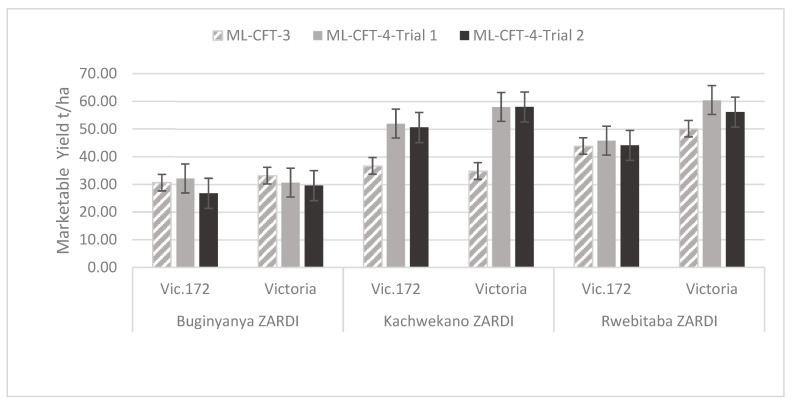
Marketable yield of transgenic event Vic.172 and the variety Victoria at each of the three locations averaged over the three trials. Error bar represents the standard error.

The genotypes performance across the three locations and two seasons showed stable yield within each location (Figure 5). Marketable yields were calculated at plant level (g/plant) but then extrapolated to t/ha using the potato planting density commonly used in Uganda. Rotten tubers were rarely encountered and were not observed to be more or less frequent by genotype or location. Yield analyses revealed significant variation across the three locations independently of the genotype. The results showed the highest number of marketable tubers at Kachwekano ZARDI whereas yield of marketable tubers was higher at Rwebitaba ZARDI (Figure 5).

The yield of marketable tubers was not significantly different (*p* > 0.05) between genotypes but significantly different (*p* < 0.05) between locations (Table 1). For the three trials (ML-CFT-3, ML-CFT-4-Trial-1, and ML-CFT-4-Trial-2), the results showed that at Buginyanya ZARDI, the average yield for Vic.172 was 29.9 t/ha and for Victoria it was 31.1 T/ha. At Kachwekano ZARDI, the average yield for Vic.172 was 46.4 t/ha, while for Victoria was 50.3 t/ha; and at Rwebitaba ZARDI, the average yield was 44.6 t/ha for Vic.172 while for Victoria, it was 55.6 t/ha. On average, yields for the second season (ML-CFT-4) were higher than those during the first season (ML-CFT-3), which matches general observations of the best rain pattern for potato cropping during the October to January season. The Genotype X location interaction was not significant (*p* > 0.05), indicating that the yield variation observed across the locations was influenced by the environment. Yield analyses produced similar results when tubers of the non-commercial category were also considered.

##### Number of Marketable Tubers of Each Genotype across Locations

**Figure 6:**
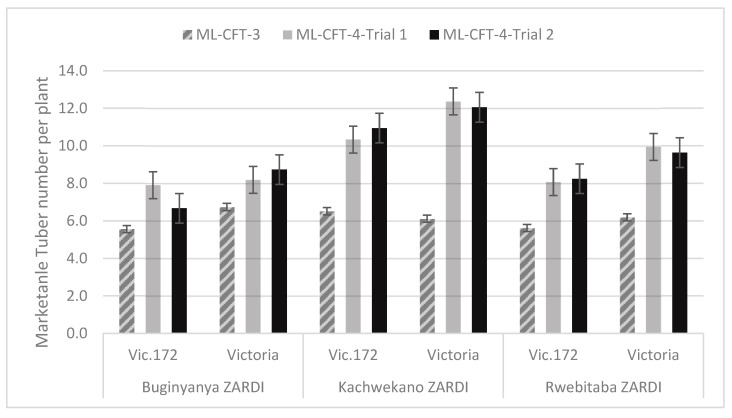
Average number of marketable tubers per plant for the transgenic event Vic.172 and the variety Victoria at the three locations. Error bar represents the standard error.

Across the three locations and two seasons, the number of marketable tubers per plant ranged from 5.6 to 12.4. The mean number of marketable tubers at Buginyanya was 6.7 for Vic.172 and 7.9 for Victoria; at Kachwekano, it was 9.3 for Vic.172 and 10.2 for Victoria, whereas at Rwebitaba, it was 7.3 for Vic.172 and 8.6 for Victoria (Figure 6).

The analysis of variance (ANOVA) on the average number of marketable tubers per plant between the transgenic event Vic.172 and the variety Victoria, and across locations, showed no statistically significant differences (*p* > 0.05) for genotypes, location, and genotype X location interaction (Table 2). When tubers of the non-commercial category were added, the comparative analysis produced the same results.

## 4. Discussion

The transgenic event Vic.172, which has complete resistance to LB, was evaluated in three locations and three seasons to assess stability of the bioengineered trait and whether it conferred any undesirable characteristics that could affect its performance if released. This was done in a systematic manner using reliable and proven methods, following internationally accepted guidelines for assessing the phenotype and agronomic performance of transgenic crops [25]. These regulatory-demanded trials permitted comparison of the transgenic event Vic.172 and the variety Victoria under identical treatments (soil, fertilizers, weeding, insecticides, and fungicides).

Resistance to LB has been a priority for potato crop improvement since the outbreak of the disease in Ireland in the1840s [3,37]. Here, we provide evidence of complete resistance to the pathogen after transferring a stack of three resistance genes into popular varieties. The transgenic event Vic.172 never presented a single lesion caused by *Phytophthora infestans* while plants of the non-transgenic variety Victoria were destroyed over a 20-day period. This 3*R*-gene stack has been introduced in other potato varieties and some have been tested in the field since 2015 [23,24]. Other *R*-gene stacks have been tested successfully in other environments with more complex pathogen populations [22]. Though stability of resistance across environments over nearly 10 years of evaluation does not guarantee the resistance will be durable in absolute terms, it is expected to be durable enough to fully control the disease long enough to establish a sustainable management of LB disease in potato based on a rotation of events with different *R*-gene stacks. The relative low diversity of the pathogen in East and Central African countries should contribute to the stability of resistance [38].

Phenotypic characterization of a transgenic event with a prospect for future release is meant to demonstrate its biosafety and to identify any undesirable characteristics that could impair its agronomic performance, environmental fitness, and consumer acceptance. Of the 13 phenotypic criteria reported here (stem color, stem wing, number of stems per tuber, number of leaflets per leaf, leaf texture, number of interjected leaves, leaf color, leaf openness, number of flowers per plant, flower color, tuber shape, tuber skin, and tuber flesh color), no phenotypic differences between the transgenic event Vic.172 and the variety Victoria were statistically significant across all locations.

Agronomic performance is always a key criterion used by farmers to decide whether or not to grow a potato variety. Tuber seed dormancy, number of sprouts per tuber, maturity duration, and yield are among the key parameters characterizing the agronomic performance of a variety. Out of the seven agronomic characteristics evaluated (sprouting, emergence, vigor, canopy height, canopy width, number of tubers per categories, and yield of marketable tubers), none of the differences between the transgenic event Vic.172 and the variety Victoria were statistically significant across locations and seasons. However, at one location, in Rwebitaba ZARDI, the difference in number of tubers and yield of marketable tubers was significant. This location produced the highest yield, which was approximately eight-fold higher than the national average yield and, thus, does not represent the low nitrogen fertilization conditions used by smallholder farmers. Overall, statistical analyses revealed that the yield variation observed across locations was not due to the genotype but was influenced by the environment.

## 5. Conclusions

The transgenic event Vic.172 displayed complete resistance to LB in the field as well as in harvested tubers. Compared to the variety Victoria, it presented few statistically significant differences across locations and seasons when both were grown under identical field conditions. Hence, this transgenic event is expected to perform as well as the variety, and receive comparable acceptance by farmers and consumers. However, additional criteria need to be evaluated before releasing it as a new variety, such as molecular characterization, compositional analysis, and an environmental risk assessment. Calculation of economic benefits indicates positive returns. Its potential for increasing productivity gain and reducing production costs is highly significant for small-scale farmers, who often lack access to sufficient fungicides to control the late blight pathogen. In Uganda, the average size of potato farms is 0.25 ha and yields average about 4 t/ha. Considering an average cost of fungicide of 60 USD/ha and an average farm-gate sale price of 150 USD/ton for potatoes, a 30% increase in productivity without fungicide costs represents a 44% profit gain. Stacking multiple *R* genes and transferring them into existing farmer and consumer -preferred varieties by genetic engineering is likely to be a sustainable and durable approach to managing LB disease, and potato diseases, such as bacterial wilt and PVY in the future. The release of such varieties will positively contribute to the environment by reducing agrochemical use and the fuel consumption/labor that spraying fungicides implies, while improving well-being by reducing risk of exposure to agrochemicals.

## Figures and Tables

**Table 1:** ANOVA for yield of marketable tubers between the transgenic event Vic.172 and the variety Victoria at all locations and between locations.

Source of Variation	d.f. ^1^	s.s. ^2^	m.s. ^3^	v.r. ^4^	F pr. ^5^
Genotype	1	129.98	129.98	2.7	0.126
Location	2	1409.71	704.86	14.65	<0.001
Genotype X Location	2	76.04	38.02	0.79	0.476
Residual	12	577.4	48.12		
Total	17	2193.13			

^1^ Degrees of freedom, ^2^ sum of squares, ^3^ mean squares, ^4^ variance ratio, ^5^ F-Value at 5% level of significance.

**Table 2:** ANOVA for marketable tuber yield per plant for the test genotypes across the three locations.

Source of Variation	d.f. ^1^	s.s. ^2^	m.s. ^3^	v.r. ^4^	F pr.^5^
Genotype	1	5.675	5.675	1.26	0.284
Location	2	18.88	9.44	2.09	0.166
Genotype X Location	2	0.105	0.053	0.01	0.988
Residual	12	54.177	4.515		
Total	17	78.837			

^1^ Degrees of freedom, ^2^ sum of squares, ^3^ mean squares, ^4^ variance ratio, ^5^ F-Value at 5% level of significance.

## Data Availability

All data are available upon request to corresponding authors.

## Supplementary Materials

The following figures and table are available online at https://www.mdpi.com/article/10.3390/biology10100952/s1. Figure S1: Tuber blight resistance assays; Figure S2: Average number of stems per planted tuber; Figure S3: Number of leaflets per leaves; Figure S4: Average number of interjected leaves; Figure S5: Flowering degree per plant in plots; Figure S6: Number of sprouts and sprout length; Table S1: ANOVA of the number of stems; Table S2: ANOVA of the number of leaflets per leaves; Table S3: ANOVA for the average number of interjected leaves; Table S4: ANOVA for flowering observed in plots; Table S5: ANOVA for number of sprouts and sprout length.
