# Supplementary material for: Comparative Phenotypic and Agronomic Assessment of Transgenic Potato with 3R-Gene Stack with Complete Resistance to Late Blight Disease
Source: Biology (Basel). 2021 Sep 23;10(10):952. doi: 10.3390/biology10100952 (PMC8533226; doi:10.3390/biology10100952)
Supplement: Supplementary file 1 [file biology-10-00952-s001.zip › biology-1352016-supplementary.pdf]

Supplementary data

A Transgenic event Vic.172 Variety Victoria

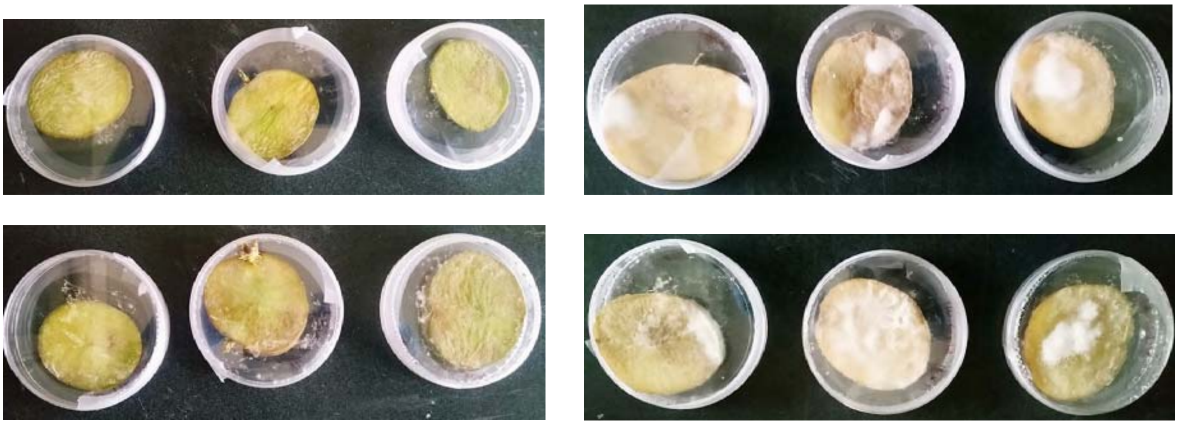

B Transgenic event Vic.172 Variety Victoria

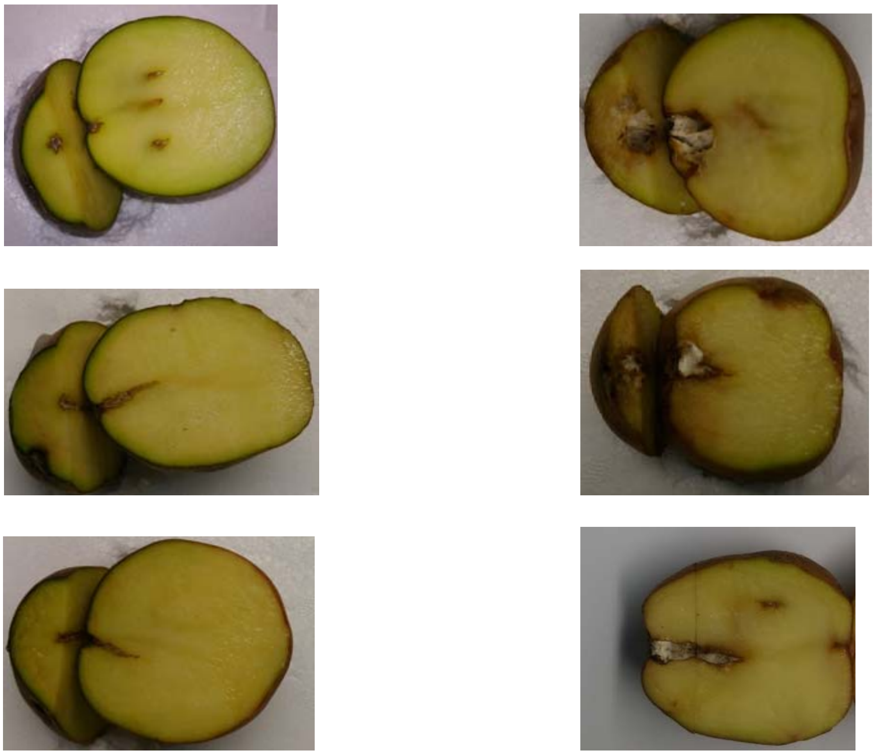

Figure S1: Tuber blight resistance assays from field-grown transgenic event Vic.172 and the variety Victoria were inoculated with *Phytophthora infestans* and evaluated 23 days post inoculation: A Tuber slices; B Half tubers infected through a hole.

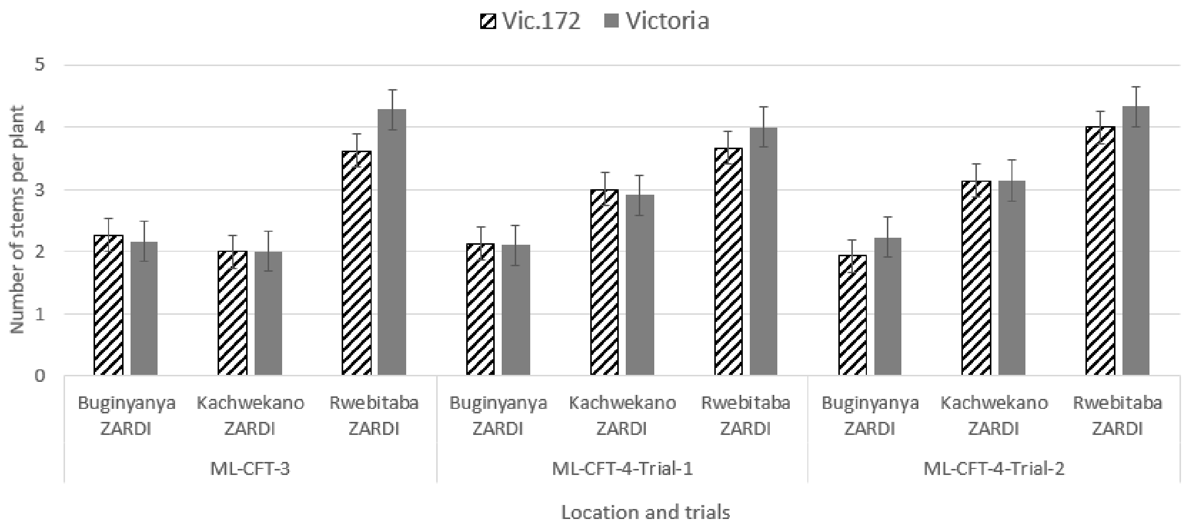

Figure S2. Average number of stems per planted tuber for the transgenic event Vic.172 and the variety Victoria at three locations during the three trials. Error bar represents the standard error.

Table S1. ANOVA of the number of stems per planted tubers for the transgenic event Vic.172 and the variety Victoria at three locations during the three trials.

**Source of variation**	**d.f.**[1]	**s.s.**[2]	**m.s.**[3]	**v.r.**[4]	**F pr.**[5]
Genotype	1	2.453	2.453	1.47	0.226
Location	2	271.644	135.822	81.34	<.001
Genotype X Location	2	4.24	2.12	1.27	0.282
Residual	438	731.346	1.67		
Total	443	1009.682			

[1] DF = Degrees of freedom, [2] Sum of squares, [3] Mean squares, [4] Variance ratio, [5] F-Value at 5% level of significance.

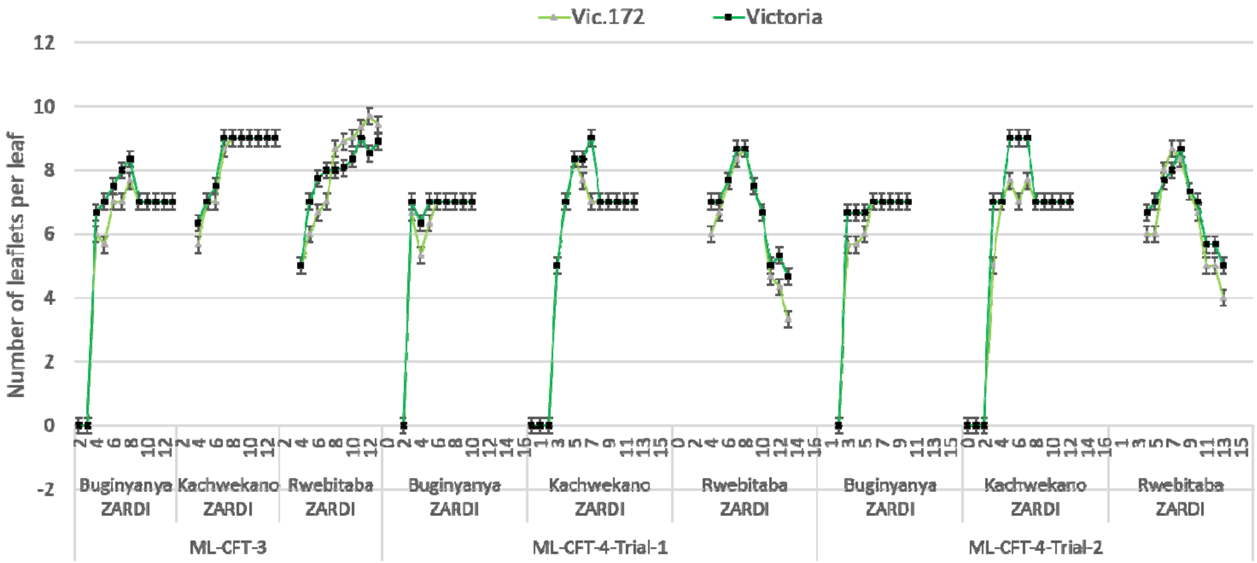

Figure S3. Number of leaflets per leaves observed at each location for the transgenic event Vic.172 and the variety Victoria during the three trials. Error bar represents the standard error.

Table S2. ANOVA of the number of leaflets per leaves observed in plots of the transgenic event Vic.172 and the variety Victoria at each location during the three trials.

**Source of variation**	**d.f.**[1]	**s.s.**[2]	**m.s.**[3]	**v.r.**[4]	**F pr.**[5]
Genotype	1	9.789	9.789	1.69	0.194
Location	2	117.507	58.754	10.15	<.001
Genotype X Location	2	0.114	0.057	0.01	0.99
Residual	606	3506.996	5.787		
Total	611	3634.407			

[1] DF = Degrees of freedom, [2] Sum of squares, [3] Mean squares, [4] Variance ratio, [5] F-Value at 5% level of significance.

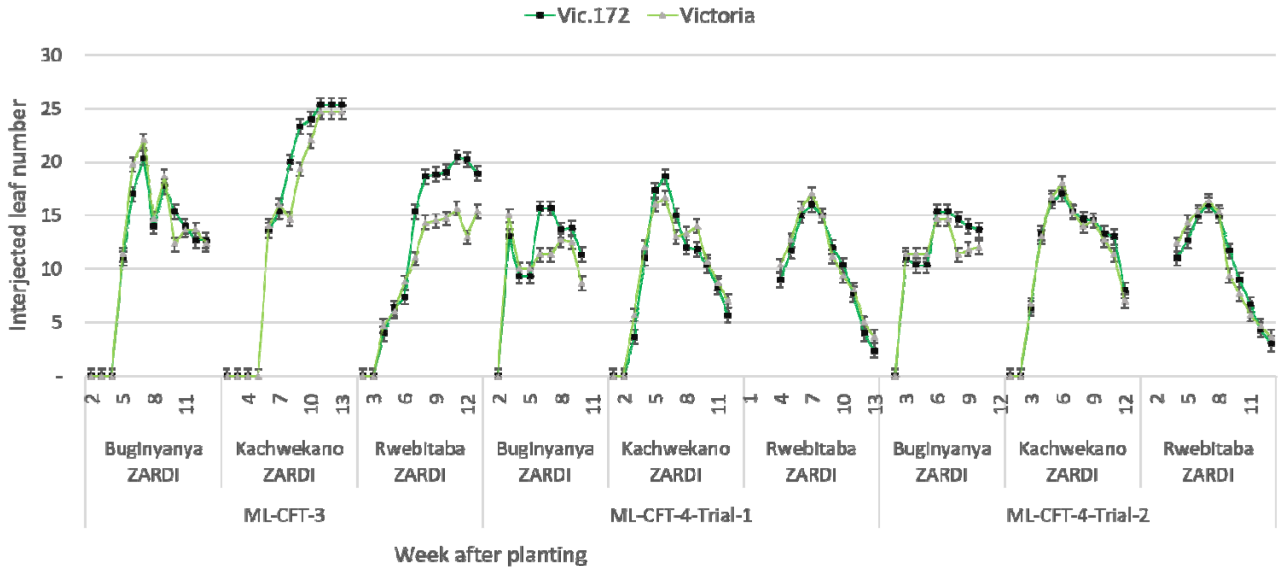

Figure S4. Average number of interjected leaves observed in plots of the transgenic event Vic.172 and the variety Victoria at each location during the three trials. Error bar represents the standard error.

Table S3. ANOVA for the average number of interjected leaves observed in plots of the transgenic event Vic.172 and the variety Victoria at each location during the three trials.

**Source of variation**	**d.f.**[1]	**s.s.**[2]	**m.s.**[3]	**v.r.**[4]	**F pr.**[5]
Genotype	1	53.25	53.25	1.23	0.267
Location	2	41.82	20.91	0.48	0.617
Genotype X Location	2	7.48	3.74	0.09	0.917
Residual	623	26927.01	43.22		
Total	628	27029.56			

[1] DF = Degrees of freedom, [2] Sum of squares, [3] Mean squares, [4] Variance ratio, [5] F-Value at 5% level of significance.

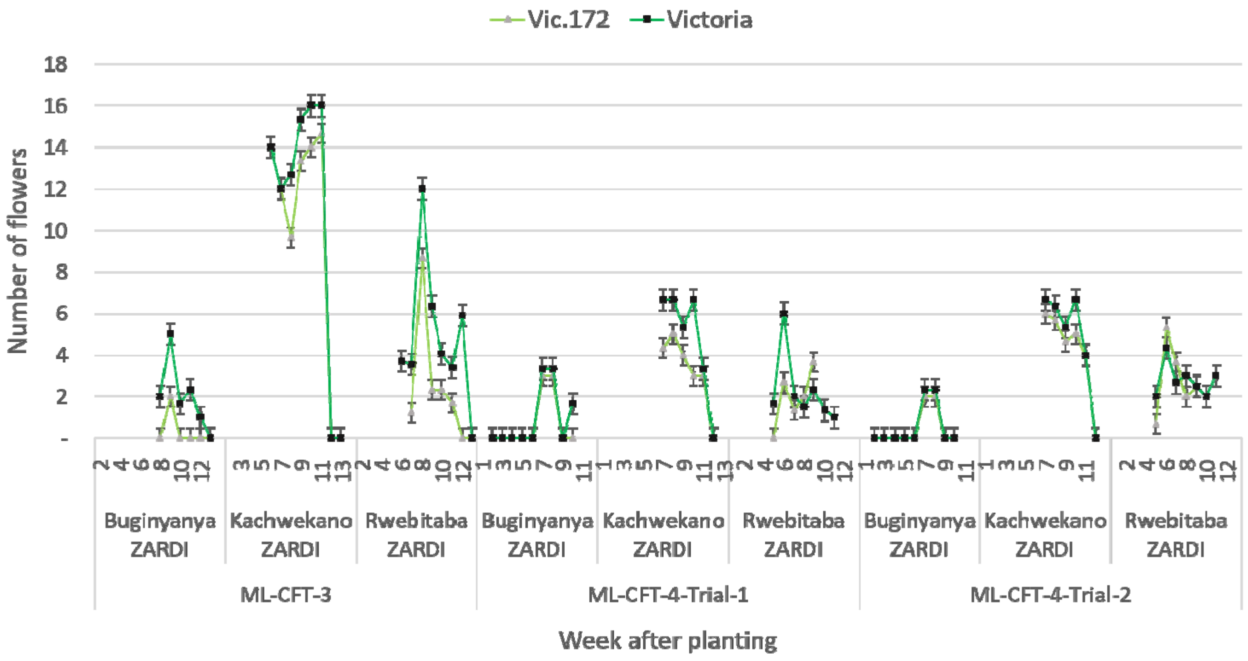

Figure S5. Flowering degree per plant in plots of the transgenic event Vic.172 and the variety Victoria at each location during the three trials. Error bar represents the standard error.

Table S4. ANOVA for flowering observed in plots of the transgenic event Vic.172 and the variety Victoria at each location during the three trials.

**Source of variation**	**d.f.**[1]	**s.s.**[2]	**m.s.**[3]	**v.r.**[4]	**F pr.**[5]
Genotype	1	4.26	4.26	0.4	0.53
Location	2	1094.63	547.31	50.83	<.001
Genotype X Location	2	14.88	7.44	0.69	0.502
Residual	231	2487.51	10.77		
Total	236	3601.27	15.26		

[1] DF = Degrees of freedom, [2] Sum of squares, [3] Mean squares, [4] Variance ratio, [5] F-Value at 5% level of significance.

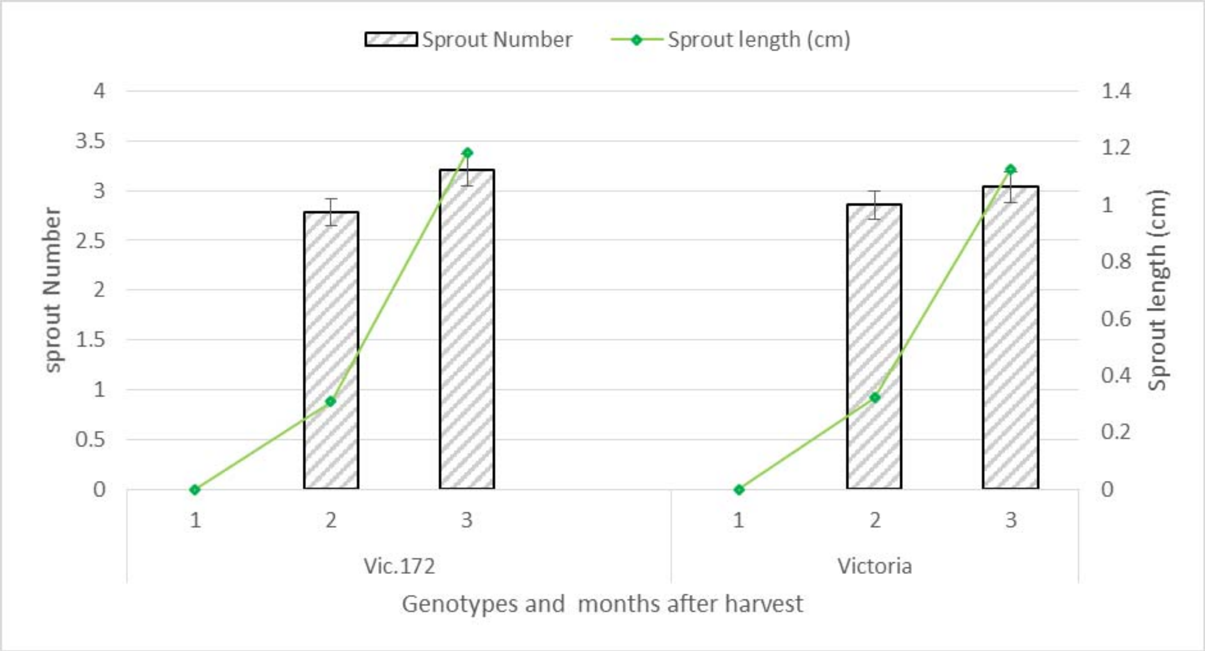

Figure S6. Number of sprouts and sprout length observed during the three months of storage before planting for the transgenic event Vic.172 and the variety Victoria. Error bar represents the standard error

Table S5. ANOVA for number of sprouts (A) and sprout length (B) for the transgenic event Vic.172 and the variety Victoria.

A

Source of variation	d.f.[1]	s.s.[2]	m.s.[3]	v.r.[4]	F pr.[5]
Genotype	1	0.054	0.054	0.02	0.895
Residual	166	512.893	3.09		
Total	167	512.946			

B

Source of variation	d.f.[1]	s.s.[2]	m.s.[3]	v.r.[4]	F pr.[5]
Genotype	1	0.0101	0.0101	0.04	0.846
Residual	166	43.9877	0.265		
Total	167	43.9978			

[1] DF = Degrees of freedom, [2] Sum of squares, [3] Mean squares, [4] Variance ratio, [5] F-Value at 5% level of significance.
